# Data Sharing as Part of the Normal Scientific Process: A View from the Pharmaceutical Industry

**DOI:** 10.1371/journal.pmed.1001936

**Published:** 2016-01-05

**Authors:** Patrick Vallance, Andrew Freeman, Murray Stewart

**Affiliations:** 1 President R&D, GlaxoSmithKline, Brentford, Middlesex, United Kingdom; 2 Head Medical Policy, GlaxoSmithKline, Brentford, Middlesex, United Kingdom; 3 Chief Medical Officer GlaxoSmithKline, Collegeville, Pennsylvania, United States of America

## Abstract

Reflecting on the WHO consultation on developing global norms for sharing data and results during public health emergencies, Patrick Vallance and colleagues argue that there are significant benefits to the principle of data sharing.

In this week’s *PLOS Medicine*, Modjarrad and colleagues report the outcome of a World Health Organisation (WHO) consultation on developing global norms for sharing data and results during public health emergencies, with a focus on clinical, epidemiologic, and genetic features of emerging infectious diseases as well as experimental diagnostics, therapeutics, and vaccines [1]. There can be little doubt that the need to find effective health care solutions as quickly as possible to prevent or stop the spread of infectious disease in an emergency makes rapid data sharing the right thing to do for patients and society. Many of the barriers to data sharing in public health emergencies identified in the paper by Modjarrad et al. have been highlighted as areas for change to enable data sharing more generally [2,3]. We are perpetually in the midst of several health care crises, including those of neglected tropical diseases and other chronic diseases, for which data sharing has the potential to lead to faster and better solutions. As a matter of principle, we should be willing to share data without regards to which disease is being studied. So, which data and when?

## Sharing Clinical Trial Data

For clinical trial data in particular, the case for sharing patient level data is compelling, whether it be to use the data from a number of studies to ask new questions and avoid waste, to aggregate data to improve the evidence base for medicine, to improve clinical trial design, or to reduce unnecessary exposure of patients to risks in potentially futile clinical trials. Experimental science rightly stresses the importance of replication, but for large clinical trials, this may not always be practical; therefore, data access is key for independent scrutiny and at least “analytical reproducibility.” There is an evolving approach to data sharing from clinical trials in which anonymized data from trials with associated metadata and supporting information are made available to other researchers following independent review of research proposals. Since May 2013, researchers have been able to access anonymized patient-level data from our clinical studies to conduct further research [4]. The system put in place has evolved to include data from a number of industry sponsors (see https://www.clinicalstudydatarequest.com). There are also other similar systems now available, with efforts now ongoing to bring all these together in a single portal. One key question is at what stage should individual patient-level data become available to bone fide researchers. Currently, for medicines aiming for approval, data release occurs following acceptance of a publication and once a regulator (e.g., European Medicines Agency [EMA]/Food and Drug Administration [FDA]) has had a chance to review the data and make a decision on approval. It may be beneficial for patient-level data availability to occur at the time of publication before there has been a regulatory decision. Such a change will require consideration on how this can be achieved without causing problems with the regulatory review process.

## Other Models of Data Sharing

Using existing data in research collaborations such as those established through the Innovative Medicines Initiative to progress new scientific projects, including projects related to health care emergencies such as Ebola (see http://www.imi.europa.eu/content/ebola-programme), is an effective means to share data. There are also other innovative models in which data can be shared to progress earlier stages of medicine discovery. For tuberculosis, malaria, and other neglected tropical infectious diseases, in addition to publishing information in the scientific literature, we have provided open access to the chemical structures and assay data for compounds that have showed significant activity in screens [5]. This has stimulated academics across the world to request compounds to conduct new research projects. Our only stipulation is that their data must also become open. For malaria, the Medicines for Malaria Ventures (MMV) have created a malaria box—available on request—that is made up of compounds and data provided by us and other research groups (see http://www.mmv.org/malariabox). This open access to early science has stimulated new discovery efforts in malaria and accelerated others, and an obvious question is whether these open approaches could usefully be applied to other areas of drug discovery [6].

## Speed of Data Dissemination and Accuracy

In emergency situations, data need to be shared quickly, before information is published. Modjarrad et al. describe an important commitment from biomedical journals that earlier disclosure will not prejudice journal publication. However, Modjarrad et al. also note a possible tension between the speed of data dissemination and its accuracy. There is a risk that premature release of incomplete or inaccurate information may mislead and delay the development of effective health care solutions. This risk is real and is true for all data release into an open environment. However, depending on the circumstances for the emergency, preliminary data could be made available with clear descriptions of the verifications that are ongoing and the remaining risks to data integrity. The challenge for the scientific community is to treat the data with appropriate caution and for journals not to allow health scares to emerge from data trawling of incomplete or provisional datasets. In stream or frequent updating of data sources presents a challenge, but not one that should be seen as a barrier to this approach.

A second aspect of data accuracy relates to data processing that needs to take place before sharing, such as anonymization. Establishing and adopting common standards and processes is going to be critically important. For example, using common clinical trial data standards will make it easier to combine data from different studies, and using agreed-upon approaches to clinical data anonymization that can be readily adopted will facilitate clinical trial data sharing. Adopting a standard approach to protecting patient privacy will be needed [7].

## Concluding Remarks

Although there are challenges, there are significant benefits to the principle of data sharing. For clinical trial data, the development and broader use of data sharing systems that are available today will help data sharing become a normal part of the clinical research process and enable more rapid and consistent data sharing when there is a public health emergency. There are existing effective models for sharing data, compounds, and knowledge to progress medicine discovery for neglected tropical infectious diseases that could be used as a basis for an approach in new emergency settings or in anticipation of future needs.

## Abbreviations

EMA: European Medicines Agency
FDA: Food and Drug Administration
MMV: Medicines for Malaria Ventures
WHO: World Health Organisation
